# Ecthyma Gangrenosum Secondary to Pseudomonal Sepsis Complicated by Acute Respiratory Distress Syndrome Following Craniotomy for Resection of a Metastasis

**DOI:** 10.7759/cureus.5543

**Published:** 2019-08-31

**Authors:** Christopher R Marcellino, Avital Perry, Christopher S Graffeo

**Affiliations:** 1 Neurological Surgery, Mayo Clinic, Rochester, USA

**Keywords:** ecthyma gangrenosum, pseudomonas, sepsis, acute respiratory distress syndrome, neurocritical care, physical examination

## Abstract

Ecthyma gangrenosum is a rare physical exam finding pathognomonic for severe bacteremia and typically associated with pseudomonal sepsis. The characteristic skin lesions appear as ring-shaped hemorrhagic pustules that evolve into necrotic ulcers. In the present case, a 62-year-old woman with a pulmonary adenocarcinoma treated with surgical resection and adjuvant chemotherapy developed three symptomatic brain masses. The lesions were presumed metastatic and initially treated with stereotactic radiosurgery; however, follow-up imaging identified treatment failure of a cerebellar lesion, and the patient was subsequently taken to surgery on an elective basis for suboccipital craniotomy and tumor resection. Although her initial postoperative course was unremarkable, on postoperative day two, she experienced a rapidly progressive neurologic and hemodynamic decline. During this period, numerous ring-shaped, necrotic cutaneous lesions rapidly appeared, consistent with ecthyma gangrenosum. In spite of multi-modality critical care treatment and resuscitation, including milrinone, multiple vasopressors, anti-pseudomonal antibiotics, and prone positioning, the patient progressed to cardiorespiratory failure and died.

## Introduction

This brief case-report focuses on the characteristic early skin lesion of ecthyma gangrenosum in a patient who developed precipitous and ultimately fatal pseudomonal sepsis with acute respiratory distress syndrome (ARDS), during routine postoperative care following elective resection of intracranial metastasis. Ecthyma gangrenosum is an important but rarely encountered physical exam finding. In addition to demonstrating an interesting and unusual finding, this case has the potential to provide readers with a valuable educational opportunity, as pseudomonal sepsis is a critical and potentially treatable entity that should be recognized and acted on whenever possible, yet one that many individuals will not directly encounter while in training.

## Case presentation

**Figure 1:**
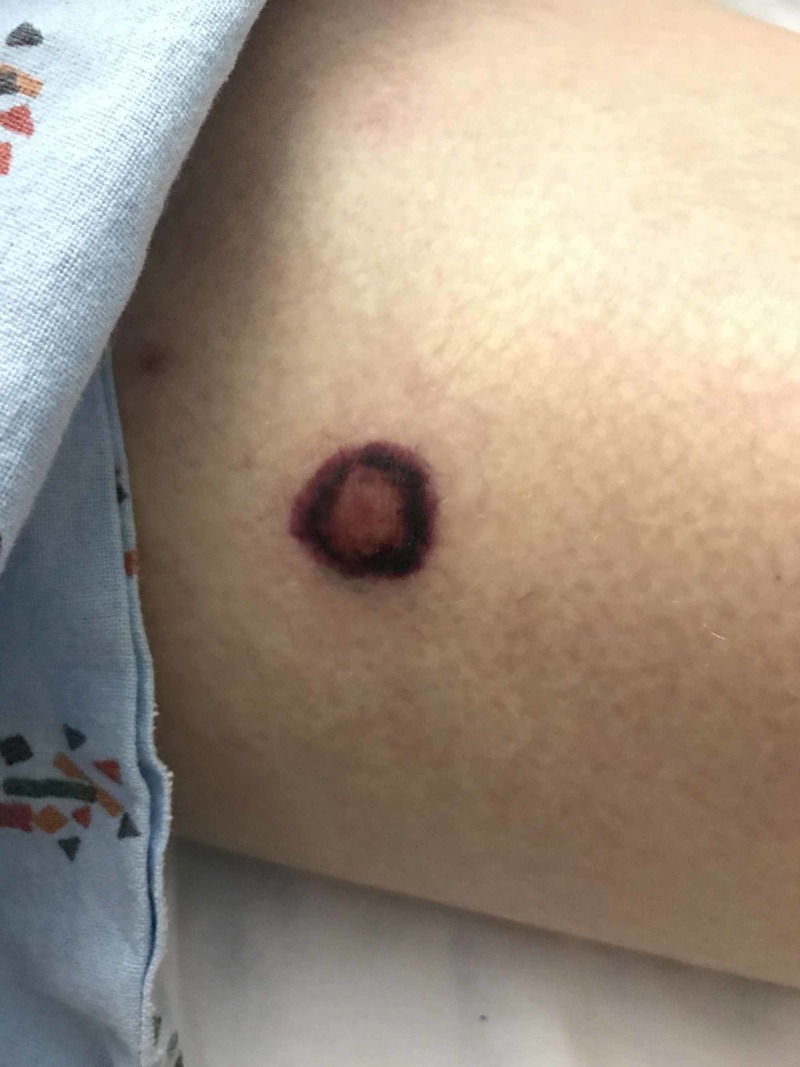
Ecthyma gangrenosum in a patient with pseudomonal sepsis

A 62-year-old woman with lung adenocarcinoma (status post-lobectomy and adjuvant chemotherapy) developed three symptomatic brain metastases, which were treated with stereotactic radiosurgery. A cerebellar lesion enlarged on surveillance imaging, and an elective suboccipital craniotomy for resection was performed, for diagnostic and therapeutic purposes (i.e., no preceding biopsy was performed). She recovered unremarkably until postoperative day two, when she experienced acute mental status changes and respiratory distress, requiring intubation. Numerous ring-shaped necrotic skin lesions appeared, consistent with ecthyma gangrenosum (Figure 1), while the patient's sepsis progressed to severe acute respiratory distress syndrome [1, 2]. Attempted treatments included broad-spectrum antibiotics, milrinone, vasopressors, and prone positioning, but the patient expired following cardiac arrest attributable to respiratory acidosis and hypoxia. Blood and sputum cultures were positive for *Pseudomonas aeruginosa*.

## Discussion

Ecthyma gangrenosum is an uncommon cutaneous manifestation of bacteremia and is essentially pathognomonic of a severe infection with *Pseudomonas aeruginosa* that has disseminated widely throughout the soft tissues. The finding almost exclusively arrises among critically ill patients, and in particular, those who are immunocompromised. Although rare case reports have identified isolated instances of *Proteus* species, *Escherichia coli*, and *Staphylococcus epidermidis*, almost all reported patients with ecthyma gangrenosum and culture data were shown to harbor *Pseudomonas aeruginosa, *warranting aggressive anti-pseudomonal treatment where the lesions are observed clinically [3-5]. 

The depicted lesion is characteristic of the painless, purpuric macules of early ecthyma gangrenosum, which appeared diffusely throughout the patient's body as her condition deteriorated. These lesions can progress to violaceous pustules, and lead to deep, infectious lesions which can necessitate surgical debridement, particularly when limited in numbers to allow for source control. Biopsy results are nonspecific, with abundant neutrophilic granulocyte and often central necrosis.

Disseminated pseudomonal infections are generally seen in immunocompromised hosts. In addition to appropriate antibiotic therapy, immune reconstitution may be necessary to treat the aggressive infectious process underlying lesions, particularly when they are diffuse. Key risk factors for the advanced disease include known antimicrobial resistance within the individual, organism, or environment (antibiogram), atypical or hospital-associated infectious sources, and rapid clinical progression; where observed, these findings may prompt an expedited institution of redundant anti-pseudomonal antibiotic coverage. The presence of frank bacteremia is the most significant predictor of an unfavorable outcome, with prior series reporting up to 96% mortality in patients with documented ecthyma gangrenosum and pseudomonal bacteremia. While ecthyma gangrenosum lesions are rarely encountered in immunocompetent patients, such individuals may experience a more indolent natural history-although mortality still exceeds 15% in non-bacteremic patients. 

In the present case, it is challenging to approximate the impact that the patient's underlying malignancy and associated treatments may have had on her acute infectious disease-independent of her immunocompromised status. Although aggressive, multi-drug regimens have shown some efficacy against resistant *Pseudomonas aeruginosa*, especially when initiated early, pseudomonal sepsis is known to be rapidly progressive in the majority of individuals. Within the context of ecthyma gangrenosum, the very high mortality observed is thought to be predominantly attributable to the high disease prevalence among immunocompromised individuals; however, poor performance status in the setting of metastatic disease likely further increases the risk of an unfavorable outcome and death. In addition to highlighting an important physical exam finding, the present case emphasizes these stakes, and the need for early recognition and very aggressive treatment whenever ecthyma gangrenosum is identified [6].

## Conclusions

In this focused, image-oriented case report, we have shown a characteristic example of ecthyma gangrenosum, highlighting an important but rarely encountered physical exam finding. Pseudomonal sepsis is a critical and potentially treatable complication that should be recognized and treated rapidly whenever possible. As the present case demonstrates, this critical complication has the potential to arise after routine operations, including elective neurosurgical procedures. Given the propensity for severe and dramatic disease progression-particularly among immunocompromised hosts-patients developing ecthyma gangrenosum should be recognized as subject to markedly increased risks of morbidity and mortality and should be treated as aggressively as possible, ideally via multi-agent anti-pseudomonal antibiotic regimens.

## References

[1] Howell MD, Davis AM (2018). Management of ARDS in adults. JAMA.

[2] Greene SL, Su WD, Muller SA (1984). Ecthyma gangrenosum: report of clinical, histopathologic, and bacteriologic aspects of eight cases. J Am Acad Dermatol.

[3] Mouna K, Akkari H, Faten H (2015). Ecthyma gangrenosum caused by Escherichia coli in a previously healthy girl. Pediatr Dermatol.

[4] Miyake S, Nobeyama Y, Baba-Honda H, Nakagawa H (2016). Case of ecthyma gangrenosum in which only methicillin-resistant Staphylococcus epidermidis was detected. J Dermatol.

[5] Firoz M, Jamal A, Ur Rehman SI (2018). Non-pseudomonal ecthyma gangrenosum and idiopathic myelofibrosis in a two-year-old girl. Cureus.

[6] Korte AKM, Vos JM (2017). Ecthyma gangrenosum. N Engl J Med.

